# IL-1β and Statin Treatment in Patients with Myocardial Infarction and Diabetic Cardiomyopathy

**DOI:** 10.3390/jcm8111764

**Published:** 2019-10-23

**Authors:** Luca Liberale, Federico Carbone, Giovanni G. Camici, Fabrizio Montecucco

**Affiliations:** 1 Center for Molecular Cardiology, University of Zürich, 8092 Schlieren, Switzerland; luca.liberale@uzh.ch (L.L.); giovanni.camici@uzh.ch (G.G.C.); 2 First Clinic of Internal Medicine, Department of Internal Medicine, University of Genoa, 16132 Genoa, Italy; federico.carbone@unige.it; 3 IRCCS Ospedale Policlinico San Martino Genoa—Italian Cardiovascular Network, 16132 Genoa, Italy; 4 University Heart Center, Department of Cardiology, University Hospital Zurich, 8001 Zurich, Switzerland; 5 Department of Research and Education, University Hospital Zurich, 8001 Zurich, Switzerland; 6 First Clinic of Internal Medicine, Department of Internal Medicine and Centre of Excellence for Biomedical Research (CEBR), University of Genoa, Genoa, University of Genoa, 16132 Genoa, Italy

**Keywords:** cardiovascular disease, myocardial infarction, diabetic cardiomyopathy, cytokines, interleukin 1β, inflammation, CANTOS, canakinumab

## Abstract

Statins are effective lipid-lowering drugs with a good safety profile that have become, over the years, the first-line therapy for patients with dyslipidemia and a real cornerstone of cardiovascular (CV) preventive therapy. Thanks to both cholesterol-related and “pleiotropic” effects, statins have a beneficial impact against CV diseases. In particular, by reducing lipids and inflammation statins, they can influence the pathogenesis of both myocardial infarction and diabetic cardiomyopathy. Among inflammatory mediators involved in these diseases, interleukin (IL)-1β is a pro-inflammatory cytokine that recently been shown to be an effective target in secondary prevention of CV events. Statins are largely prescribed to patients with myocardial infarction and diabetes, but their effects on IL-1β synthesis and release remain to be fully characterized. Of interest, preliminary studies even report IL-1β secretion to rise after treatment with statins, with a potential impact on the inflammatory microenvironment and glycemic control. Here, we will summarize evidence of the role of statins in the prevention and treatment of myocardial infarction and diabetic cardiomyopathy. In accordance with the dual lipid-lowering and anti-inflammatory effect of these drugs and in light of the important results achieved by IL-1β inhibition through canakinumab in CV secondary prevention, we will dissect the current evidence linking statins with IL-1β and outline the possible benefits of a potential double treatment with statins and canakinumab.

## 1. Introduction

Statin discovery dates back to 1976, when mevastatin was isolated from cultures of *Penicillium citrinum* and proven to inhibit the production of cholesterol molecules [1]. Further experiments showed that statins occupy a portion of the rate-controlling enzyme of cholesterol synthesis 3-hydroxy-3-methylglutaryl-CoA (HMG-CoA) reductase (HMGR) by binding its active site with very high affinity, thus displacing the natural substrate, HMG-CoA, and inhibiting its function [2]. Furthermore, the statin-related reduction of circulatory lipoprotein induces the hepatic expression of low-density lipoprotein (LDL) receptor (LDLR) and LDL clearance from the bloodstream, thus accounting for a further decrease in circulating cholesterol levels [3]. Thanks to this dual mechanism of action and a good safety profile, both natural and synthetic statins became, over the years, the first-line therapy for dyslipidemia patients and a real cornerstone of cardiovascular (CV) preventive therapy. Soon after first trials with statins were published, evidence suggested that those compounds might have putative, non-lipid-related effects. Both Cholesterol and Recurrent Events (CARE) and Long-Term Intervention with Pravastatin in Ischaemic Disease (LIPID) trials showed that their overall cardiovascular benefit was disproportionate to the magnitude of lipid reduction [4,5]. In addition, the speed by which statins exercised their protective role was faster than that obtained with other lipid-lowering interventions such as ileal bypass [6]. These “pleiotropic” effects have been related to statins’ inhibitory effect on the activation of different intracellular signaling mediators downstream the mevalonate pathways (i.e., Rho, Ras, and Rac proteins) alongside direct stimulatory effects on peroxisome proliferator-activated (PPAR)-α and -β [7].

Lipids and inflammation are closely interconnected and contribute to the pathogenesis of most CV disease [8,9]. Among those, myocardial infarction constantly rates among the most important causes of morbidity and mortality worldwide, while diabetic cardiomyopathy is an emerging disease whose incidence is set to rise in the next years following the increased prevalence of the diabetic population. Although the role of circulating lipoproteins in the determination of the individual CV risk have been appreciated since a long time ago, recently, clinical and experimental observations support a role for systemic inflammation [10]. Inflammatory cells and cytokines have been identified in human atherosclerotic vessels, and their dynamic regulation plays an important role in cardiac remodeling [11,12]. Observational studies reported a reduced CV risk in patients being treated with anti-inflammatory agents for immunological disease (e.g., rheumatoid arthritis), supporting the concept of inflammation as a valuable target for CV prevention [13]. However, not all anti-inflammatory drugs provided efficacy in reducing CV risk as different trials designed to test this hypothesis gave negative results (i.e., Cardiovascular Inflammation Reduction Trial [CIRT] testing methotrexate), and non-steroidal anti-inflammatory agents are even associated with an increased CV morbidity [14,15]. Of importance, in 2017, the Canakinumab Anti-Inflammatory Thrombosis Outcomes Study (CANTOS) trial showed the efficacy of IL-1β neutralization in patients with established coronary heart disease, highlighting this cytokine and its pathway as effective targets, as well as suggesting that specific interaction with inflammatory mediators might be a better strategy than providing anti-inflammation in a global fashion [16,17].

In this review article, we aim to summarize evidence of the role of statin treatment in myocardial infarction and prevention of myocardial remodeling in patients with diabetes mellitus. In accordance with the dual lipid-lowering and anti-inflammatory effect of these drugs and in light of the important results achieved by CANTOS in CV secondary prevention, we dissect the current evidence linking statins with IL-1β and outline possible benefits of double treatment with canakinumab.

## 2. Statins in Myocardial Infarction and Diabetic Cardiomyopathy 

### 2.1. Myocardial Infarction

Coronary atherosclerotic heart disease is the major cause of CV events including stable and angina, non-ST-segment elevation myocardial infarction (NSTEMI), ST-segment elevation myocardial infarction (STEMI), and sudden coronary death [18]. Ample evidence demonstrated the key role of dyslipidemia and, in particular, of elevated LDL levels in the development of coronary heart disease and thus CV risk. As such, statins have become the first-line therapy for hyperlipidemia and reduction of CV risk in patients at high and very high risk [19]. With clinical guidelines becoming increasingly stringent with respect to cholesterol levels [20,21,22], the prescription and utilization of statins in the last 30 years has increased considerably, with most of the patients taking these drugs for primary prevention of CV events [23]. To date, several randomized clinical trials (RCTs) and systematic reviews have investigated the role of statins in this setting, reaching different conclusions [24,25,26,27]. These apparent discrepancies might be explained by different factors: (i) the population in primary prevention is highly heterogeneous, including patients with low CV risk and those with chronic kidney disease or diabetes mellitus with organ damage, who are usually considered as risk-equivalent to CV patients; (ii) most published systematic reviews, although focusing on primary prevention, included trials in which a proportion of patients had a history of CV disease. A very recent overview of systematic reviews tried to overcome these limitations by including exclusively primary prevention trials or individual patient data of trial participants using only data from patients without established CVD [28]. Here, the authors report a trend towards reduction of all-cause mortality in all systematic reviews, although this reached statistical significance only in one study out of three [28]. Furthermore, when patients where stratified for baseline risk, the effect of statin treatment lost statistical significance in almost all categories [28]. Similar inconclusive results were reported also when considering different outcomes such as vascular or non-vascular deaths or composite ones; here, again, stratification for baseline risk deeply impacted the magnitude of the results. The authors concluded that despite the high number of patients under statins treatment for primary CV prevention, the evidence for their prescription in this setting is very limited and should be substantiated by a careful individual assessment of baseline risk, absolute risk reduction, and potential harm [28].

On the other hand, the role of high-intensity statin treatment as a secondary prevention measure to reduce the recurrence of CV and cerebrovascular events is well established and highlighted by all international guidelines [20,21,22]. In patients with previous myocardial infarction and stroke, statins blunt the rate of recurrent CV events as well as the need for revascularization procedures. In addition, mortality is considerably reduced: In the five years after myocardial infarction, treatment of only 30 patients with statin is already able to prevent one cardiovascular death [29]. The pioneering Scandinavian Simvastatin Survival Study (4S) trial compared simvastatin treatment vs. placebo in *n* = 4444 patients with angina pectoris or previous myocardial infarction and found statin to greatly reduce the risk of death (both cardiovascular and non-cardiovascular ones) as well as that of undergoing revascularization procedures [29]. More recently, the Pravastatin or Atorvastatin Evaluation and Infection Therapy—Thrombolysis in Myocardial Infarction 22 (PROVE-IT TIMI 22) trial compared high-intensity (atorvastatin 80 mg/day) vs. moderate-intensity (pravastatin 40 mg/day) statin treatment early after ACS and found the strongest intervention to bring an additional 16% reduction of cardiovascular events as compared to pravastatin 40 mg/day, in 4162 patients [30]. Of interest, the benefit was already evident within 30 days and became statistical significant throughout the 2.5 years of follow-up [30]. After this, several other trials tested different statins at different dosages, and results have been summarized in numerous meta-analyses. Among them, in 2010 the Cholesterol Treatment Trialists (CTT) Collaboration analyzed five randomized trials comparing more intensive vs. less intensive statin regimens in *n* = 39612 patients with ACS or stable coronary disease [30]. As a result, the intensive statin treatment showed a greater reduction in major CV events compared to the less intensive one. Moreover, this research highlighted that statin benefit is maintained among patients with and without hypercholesterolemia, and no threshold was found under which LDL lowering was ineffective [30]. Recently, a specific analysis investigated specific population such as elderly people and the benefit of statin treatment in secondary prevention remained valid, although some warnings have been raised for specific high-dose regimens [31,32]. Importantly, the prognostic role of LDL reduction and the importance of an early start to high-dose statin treatment after ACS was consistently shown among the majority of clinical trials. This aspect has been taken further by the recent secondary prevention trials investigating the use of non-statin lipid-lowering agents in association with the maximally tolerated statin dose in ACS patients, confirming the concept of “the lower, the better” [33]. Accordingly, the very recent guidelines on dyslipidemia by the European Society of Cardiology (ESC)/European Atherosclerosis Society (EAS) have adopted a more aggressive approach with never-seen-before very low targets for LDL levels in high-risk categories (such as individuals with previous CV events) [20]. Indeed, the LDL target for patients at very high risk is now set at 1.4 mmol/L (<55 mg/dL), while in patients at very high-risk with multiple recent events, the target reaches 1.0 mmol/L (<40 mg/dL) [20].

### 2.2. Diabetic Cardiomyopathy

Diabetic patients are at increased risk of developing heart failure. The Framingham Heart Study clearly indicated that diabetes and heart failure are associated, independently of the presence of coronary artery disease and hypertension [34]. As such, hyperglycemia can cause cardiac insufficiency not only by increasing the risk of heart failure determinants but also by directly affecting cardiac structure and function. Diabetes is associated with cardiac oxidative stress, intracellular ion abnormalities, inflammation, and mitochondrial dysfunction, with metabolic turbulences directly causing the development of heart failure, and particularly, heart failure with preserved ejection fraction, by altering specific signaling pathways [35]. Although debated [36], diabetes is also thought to be associated with systolic heart failure, as previous work showed that indexes of systolic function may be slightly reduced in diabetic patients without overt coronary disease [37,38]. In this case, the chronic alteration of glucose levels may cause a reduction of myocardial flow reserve due to microvascular alterations and lead to subendocardial ischemia and systolic dysfunction [39]. Although the molecular signaling deranged by the chronic exposure to high glucose levels is very diverse and several pathways have been involved in the pathophysiology of diabetic cardiomyopathy, in general, they all converge towards the activation of the transcription factor NF-kB, which then leads to upregulation of cytokines, chemokines, and adhesion molecules [40]. Indeed, genetic or pharmacologic inhibition of this nuclear factor mitigates cardiac inflammation and oxidative stress in animal models of diabetes, thus preventing the development of diabetic cardiomyopathy [41,42]. Glycemia-oriented therapy does not effectively prevent cardiac complications of long-term type 2 diabetes mellitus [43], thus other drugs have been tested to reduce cardiac damage. Statins have been hypothesized to hold a protective role in the setting of diabetic cardiomyopathy thanks to their anti-inflammatory role. Furthermore, hyperlipidemia is associated with intracardiac accumulation of fatty acids and dysfunction due to lipotoxicity in the diabetic myocardium [44]. Pre-clinical evidence strongly supports this hypothesis; atorvastatin could improve left ventricular function by reducing cardiac intramyocardial inflammation and myocardial fibrosis in an experimental model of diabetic cardiomyopathy [45]. In addition, atorvastatin could reduce β-adrenergic dysfunction in rats with diabetic cardiomyopathy via increasing nitric oxide (NO) availability [46]. Rosuvastatin also exhibited protective properties in this setting by reducing NLRP3 inflammasome and IL-1β activation via suppression of MAPK pathways [47]. Finally, simvastatin could reduce cardiac dysfunction in streptozotocin-induced diabetic rats by attenuating hyperglycemia-induced cardiac oxidative stress, inflammation, and apoptosis. In the clinical setting, intensive lipid control with statins and other drugs is associated with an important decrease of cardiovascular risk in diabetic patients [48,49,50]. Accordingly, statins together with other lipid-modifying agents (i.e., peroxisome proliferator-activated receptor (PPAR) agonists) are suggested by diabetes guidelines for both primary and secondary CV prevention [51]. This being said, statins failed to effectively modify the course of diabetic cardiomyopathy, and they may even facilitate the onset of diabetes by impacting peripheral insulin sensitivity and β-cell function [52]. Nevertheless, discontinuing statin therapy in diabetic patients is not recommended [53].

## 3. Statins, Inflammation, and IL-1β

The effectiveness of statin anti-inflammatory properties in the CV setting has been definitively proven in clinical trials. The Justification for the Use of Statins in Prevention: an Intervention Trial Evaluating Rosuvastatin (JUPITER) trial enrolled apparently healthy persons without hyperlipidemia but with elevated hs-CRP and demonstrated that treatment with rosuvastatin significantly reduced the incidence of major CV events [54]. Similarly, the Pravastatin Or Atorvastatin Evaluation and Infection Therapy (PROVE-IT), Aggrastat to Zocor (AtoZ), and Improved Reduction of Outcomes: Vytorin Efficacy International Trial (IMPROVE-IT) trials also reported the clinical relevance of statin-related hs-CRP reduction [30,55,56]. Statins exert these anti-inflammatory effects by blunting the downstream synthesis of molecules in the mevalonate pathway through the inhibition of small GTPase prenylation and isoprenoid production [57,58,59]. Of note, small GTPases regulate different signaling pathways and thus cellular processes dependent on isoprenylation and involved in the development of CV diseases [60,61]. Rho and Rac cooperate in the surge of oxidative stress and inflammatory mediators that characterize different pathologic processes [62,63]. Furthermore, Ras is thought to play a central role in the regulation of cellular growth and proliferation [64]. The inhibition of those pathways is associated with a number of protective immunomodulatory effects in inflammatory cells and vascular and myocardial tissue [65,66]. In vascular cells, statins can enhance the availability of protective nitric oxide (NO) by increasing its synthesis and reducing its degradation [67,68]. This accompanies a sensible reduction of endothelial oxidative stress and modulation of redox-sensitive transcription factors such as NF-kβ and activator protein 1 (AP-1), key enzymes involved in the regulation of several pro-inflammatory genes [69]. As a result, treatment with statins is associated with blunted expression of pro-thrombotic factors as well as different adhesion molecules such as vascular cell adhesion molecule 1 (VCAM-1), platelet endothelial adhesion molecule 1 (PECAM-1), intercellular cell adhesion molecule-1 (ICAM-1), and P-selectin [70,71,72]. Moreover, statin treatment reduces monocyte, endothelial, and vascular smooth cell production of different pro-inflammatory cytokines including monocyte chemoattractant protein-1 (MCP-1), regulated on activation, normal T cell expressed and secreted (RANTES), and interleukin (IL)-6 and IL-8 [70,73,74]. Statins also exert their immunomodulatory effects by reducing monocyte expression of CD11β (an integrin with a key role in monocyte–endothelium interaction), suppressing the expression of major histocompatibility complex (MHC) class II protein, as well as reducing the proliferation and differentiation of activated T- and B-lymphocytes [75,76,77].

**Figure 1:**
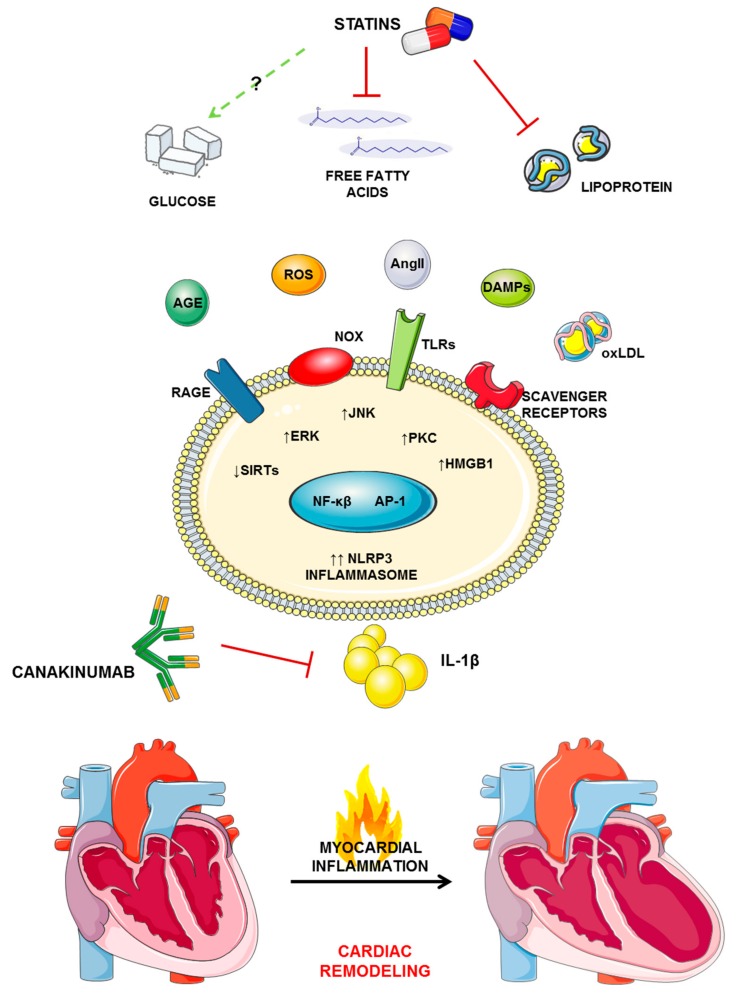
Altered lipid and glucose metabolisms share common molecular pathways in the pathophysiology of cardiac remodeling, relying on the release of pro-inflammatory interleukin (IL)-1β. The increased levels of different deleterious mediators (such as AGE, AngII, DAMP, and modified lipoproteins) are sensed by promiscuous receptors on cell surfaces and trigger secondary signaling pathways leading to activation of NF-κβ and AP-1, two transcription factors involved in the regulation of NLRP3 inflammasome activity. The activated inflammasome then leads to the activation and release of IL-1β, which fuel the sterile inflammation associated with cardiac remodeling. AGE: advanced glycation end-product; AngII: angiotensin II; AP-1: activator protein 1; DAMPs: damage-associated molecular patterns; ERK: extracellular signal-regulated kinases; HMGB1: high mobility group box 1; IL-1β: interleukin 1β; JNK: Janus kinases; NF-κβ: nuclear factor kappa-light-chain-enhancer of activated B cells; NLRP3: NACHT-, LRR-, and PYD-domain-containing protein 3; NOX: NADPH oxidase; PKC: protein kinase C; TLR: Toll-like receptor; RAGE: receptor of AGE; ROS: reactive oxygen species; oxLDL: oxidized low-density lipoproteins.

Dyslipidemia, altered glucose metabolism, and inflammation share several cardiac signaling pathways and are closely interconnected (Figure 1). Although the anti-inflammatory role of statins is widely accepted, different studies demonstrated that those molecules could paradoxically increase the production of IL-1β—among the most important pro-inflammatory cytokines—as a result of reduced protein prenylation in immune cells [78]. Mature, active IL-1β derives from the cleavage of its pro-form by the NOD-like receptor family, pyrin-domain-containing (NLRP) 3 inflammasome [79]. The NLRP3 inflammasome complex is formed by the sensor molecule NLRP3, the adaptor protein ASC, and pro-caspase-1. This multimeric protein complex regulates the release of cytokines IL-1β and IL-18, alongside initiating an inflammatory form of cell death known as pyroptosis [80]. Inflammasome activation is a two-step process that requires adequate priming of NLRP3 followed by a signal triggering the assembling [80]. The priming step occurs via different inflammatory stimuli such as TLR4 agonists, resulting in activation of NF-kB and transcription of NLRP3 and pro-IL-1β [81,82,83]. Furthermore, NLRP3 priming also associates with post-translational modifications of NLRP3 (such as phosphorylation and ubiquitination), further regulating its activation [84,85]. The second step is provided by the recognition of damage-associated molecular patterns (DAMPs) causing the perturbation of cellular metabolism with the production of reactive oxygen species, ion disturbances, and lysosomal disruption [86,87,88,89]. Due to its pro-inflammatory role, the NLRP3 inflammasome has progressively become an important molecular target to cope with different chronic diseases, including myocardial infarction and diabetes [90]. To date, five specific NLRP3 inhibitors have been validated in vivo or in vitro and entered clinical testing at different phases [91]. On the other hand, many drugs able to modify cellular metabolism and homeostasis have been shown to activate the inflammasome [92]. Although some controversies still exist due to possible differences between different molecules, statins are acknowledged among NLRP3 activators and thus IL-1β inducers. Of note, this characteristic is thought to account for their association with diabetes onset [78,93,94]. Various statins have been shown to increase IL-1β secretion from macrophages through NLRP3 activation, but none of them were able to act as a priming agent on the inflammasome as they all need bacterial liposaccharide to induce caspase-1-dependent cleavage of pro-IL1β into its active form [95,96]. In a report from 2014, Henriksbo and colleagues showed that long-term treatment of obese mice with fluvastatin promoted insulin resistance in adipose tissue and increased caspase-1 activity and IL-1β production in adipose tissue explants in the presence of LPS [95]. Of interest, this effect was not observed in NLRP3^−/−^ explants and was reversed by glyburide, a known inflammasome inhibitor and antidiabetic drug [95]. Similarly, fluvastatin could increase the secretion of 1L-1β and IL-18 in peripheral blood mononuclear cells stimulated by *Mycobacterium tuberculosis* [97]. In additon, lovastatin increases reactive oxygen species (ROS) and synergizes with LPS to trigger IL-1β release in macrophages and monocytes [98]. Of interest, these pro-inflammatory effects have been shown to relate with statin-related disturbances on protein prenylation as addition of mevalonate or GGPP—an intermediate in the mevalonate pathway—could prevent IL-1β release [96,99]. Recently, this aspect has been further dissected by showing that, differently from LDL lowering, statin-related reduction of isoprenoids was required for NLRP3/caspase-1 inflammasome activation and IL-1β-dependent insulin resistance in adipose tissue [100]. Furthermore, supplementation of geranylgeranyl isoprenoids or caspase-1 inhibition could prevent statin-induced alteration of insulin signaling [100]. Moreover, IL-1β, but not IL-18, is necessary to induce insulin resistance in adipose tissue treated with atorvastatin [100]. Summarizing, inflammasome activation and IL-1β secretion likely link statins with impaired glucose metabolism. Thus, inflammasome might be an effective molecular target to reduce statin-related diabetes onset. On the other hand, targeting the inflammasome and IL-1β might reduce the effectiveness of statin treatment on CV prevention, as the importance of this interleukin has been recently highlighted in the CANTOS trial.

## 4. Perspective

Human coronary plaques are inflammatory lesions in which immune cells and inflammatory molecules are detectable at a high level and play pivotal roles [101,102,103,104]. Recently, the CANTOS trial confirmed the inflammatory theory of atherosclerosis and shed new light on the role of IL-1β in CV risk determination [16]. A total of 10,061 patients with a previous myocardial infarction and showing inflammatory residual risk (CRP > 2 mg/L) under optimal CV-protective therapy were enrolled in this randomized, double-blind trial to receive canakinumab, a IL-1β inhibitory monoclonal antibody, or a placebo every 3 months. Levels of lipids remained unaltered upon treatment with canakinumab, while a significant decrease in CRP levels was observed already after the first administration of the anti-inflammatory drug. The primary endpoint composed of cardiovascular death, non-fatal myocardial infarction, and non-fatal stroke was successfully met by the intermediate doses of the drugs (100 and 150 mg/administration) [16]. Of interest, those patients with CRP levels reduced to <2 mg/L after the first administration benefitted the most from the long-term treatment as this was associated with a 31% reduction in CV mortality, a 31% reduction in all-cause mortality, and a 25% reduction in major adverse CV events [105]. Conversely, in patients with on-treatment high-sensitivity CRP ≥ 2 mg/L, the treatment effects were non-significant [105]. Of interest, canakinumab was also effective in reducing rates of non-cardiovascular inflammatory disease, such as lung cancer, arthritis, and gout. As expected, patients treated with IL-1β inhibitory antibody had a higher rate of fatal infections as well as of neutropenia or thrombocytopenia [16]. The CANTOS trial not only provided solid proof of the effectiveness of IL-1β inhibition in secondary CV prevention; it also allowed a deepening of the complex relationship between lipids and inflammation. Indeed, cholesterol crystals can induce IL-1β activation via canonical (i.e., NLRP3-mediated) and non-canonical pathways, then IL-1β establishes a vicious circle that ends up in further pro-IL1β cleavage [106,107,108]. Then, blocking this pathway via canakinumab could reduce the effect of lipids on atherosclerotic inflammation. On the other hand, substantial evidence exists demonstrating a role for inflammation on the induction of dyslipidemia [109,110]. Differently from other anti-inflammatory drugs, in CANTOS, canakinumab did not affect cholesterol levels, while it slightly increased triglycerides [16]; thus, the cardiovascular preventive effects did not depend on any lipid effects related to IL-1β.

Canakinumab is effective and relatively safe for secondary prevention of CV events; whether this might also be the case for primary CV prevention or for the treatment of myocardial infarction sequelae such as cardiac remodeling remains to be investigated. Cardiac repair after myocardial infarction depends on the tight regulation of sterile inflammation, which serves to clear damaged cells and promote the formation of a functional scar; alterations of the inflammatory balance associate with deleterious myocardial remodeling, resulting in cardiac dysfunction and heart failure [111]. Being a key regulator of inflammation, IL-1β plays an important role in orchestrating the inflammatory response in an ischemic/reperfused myocardium [112]. In this setting, IL-1β can activate downstream mediators which further amplify inflammation via MAPK and NF-κB signaling. Furthermore, it allows for the spatial extension of inflammation by activating local parenchymal and infiltrating cells that express its receptor and facilitates leukocytes recruitment via increasing the expression of adhesion molecules and chemoattractant in the damaged myocardium [111]. In line with this evidence, previous experimental studies reported a role of IL-1 blockade in preventing adverse cardiac remodeling (Table 1).

Unfortunately, the few randomized clinical trials and observational and cohort studies that have evaluated the effect of IL-1β inhibition in relation to the development of post-MI cardiac remodeling have provided conflicting results [121,122,123,124] (Table 2). Of interest, most of them were based on the unspecific blockage of IL-1 receptor, which recognizes both IL-1α and β isoforms; whether a more specific targeting of the IL-1β pathway via canakinumab might provide additional beneficial effects on top of statins in the context of post-myocardial remodeling remains to be fully determined. Recently, a sub-analysis of the previously mentioned CANTOS trial suggested post-MI treatment with canakinumab to dose-dependently reduce hospitalization for heart failure and the composite of hospitalization for heart failure or heart-failure-related mortality as compared to a placebo [125]. In this regard, it is important to take into consideration that the CV-protective role of canakinumab has been demonstrated only in patients with residual inflammatory risk, while that of statins is not restricted to this group. Furthermore, lipid-lowering therapies hold a very competitive risk/benefit balance even when very low LDL levels are reached [126], while this is not the case for IL-1β blockade, which is associated with a higher risk of sepsis and fatal infections.

Similarly, given the relevance of inflammatory mediators, and particularly IL-1β, in the pathophysiology of diabetes and diabetic cardiomyopathy [127], targeting the NLRP3/IL1β pathway could effectively reduce the burden of this disease. Given the high number of diabetic individuals under cardio-protective treatment with statins and the possible deleterious effect of these drugs on Il-1β activation and thus glycemic control, adding IL-1β inhibition on top of statin treatment might give additional benefit in terms of CV protection. In this sense, it will be very important to understand whether the inflammasome could be safely targeted without altering the general anti-inflammatory effect of statins. How statins could be generally anti-inflammatory and thus protective in the CV setting while increasing the risk of diabetes remains to be fully explained. In other words, on which pathophysiological aspect of the two diseases does the mechanism of action of statins differ? Different investigators have previously tried to address this question, with different hypotheses being made [78]. Statins might have different effects on different cells with different roles in the diseases. In this sense, the effect of statins on endothelial cells should drive the protective CV effects, while their roles on adipocytes, pancreatic islet cells, or myocytes could be of more relevance in diabetes onset and diabetic cardiomyopathy [78]. In addition, the cholesterol-lowering effect might play a more important role in the prevention of CV disease as compared to that played in diabetes development. The connection between statins, NLRP3/IL-1β, and insulin resistance remains to be characterized in depth, and many mechanistic questions are still unsolved; understanding these aspects might pave the way for new therapeutic strategies, including a combination of statins and IL-1β inhibition.

## 5. Conclusions

Firstly introduced to reduce circulating LDL, statins soon became pillars of prevention and treatment of CV diseases. Aside from their lipid-lowering actions, statins hold different pleiotropic effects that are thought to deeply contribute to their CV-protective effect and involve the modulation of the inflammatory response. Statins are recommended for prevention of myocardial infarction in patients with dyslipidemia, high, or very high cardiovascular risk. In diabetic subjects, statins have been hypothesized to reduce the development of diabetic cardiomyopathy thanks to their anti-inflammatory effect. Despite the encouraging results of the pre-clinical tests, statins failed to effectively modify the course of heart failure in diabetic patients and may even facilitate the onset of diabetes in patients without previous glucose disturbances. Thanks to the CANTOS trial involving the IL-1β inhibitory antibody canakinumab, this pro-inflammatory cytokine has recently emerged as an effective and relatively safe target for secondary CV prevention in patients with residual inflammatory risk. Although generally seen as anti-inflammatory drugs, statins may have different effects on IL-1β synthesis in different cells, with some studies even demonstrating a paradoxical increase. In consideration of the detrimental role of IL-1β in the pathophysiology of myocardial infarction and diabetic cardiomyopathy, adding canakinumab on top of statins in these patients might then provide a stronger inhibition of the IL-1β-mediated inflammatory response, with additional beneficial effects on the pathophysiology of these diseases. In addition, in patients treated with statins, canakinumab might even be able to reduce statin-induced insulin resistance as this is thought to depend on the activation of the NLRP3 inflammasome/IL-1β pathway. Further specific investigations will be needed to test this hypothesis in order to reduce the very high global burden of myocardial infarction and diabetic cardiomyopathy.

## Figures and Tables

**Table 1:** Experimental studies investigating the effect of IL-1 inhibition in preventing cardiac remodeling after myocardial infarction.

Author	Year	Drug(dose)	Schedule	Results
Abbate A. et al. [113]	2008	Anakinra (1 mg/kg)	Immediate or delayed (24 h after ischemia) and then daily for 6 days.	Anakinra-treated mice showed signs of more favorable ventricular remodeling.
Van Tassell et al. [114]	2010	IL-1 Trap (1, 5 or 30 mg/kg)	Every 48 h after surgery.	Mice treated with 5 or 30 mg/kg of IL-1 Trap had more favorable cardiac remodeling and echocardiographic assessment of infarct size at 7 days.
Toldo et al. [115]	2012	rhIL-1Ra	10 mg/kg given either 30 min or 4 h prior to surgery	Irrespective of dose, treated mice showed marked cardio-protection in terms of LVEF and the reduction of the infarct size.
Toldo et al. [116]	2013	Anti-IL-1β Ab	10 mg/kg immediately after surgery and then 1 week later.	When compared with control vehicle, anti-IL-1β Ab limit left ventricular enlargement and improve systolic dysfunction by inhibiting cardiomyocyte apoptosis.
Toldo et al. [117]	2014	Anti-IL-1β Ab	10 mg/kg 1 week after surgery and then weekly for 9 weeks.	After 10 weeks, anti-IL-1β Ab prevents reduction of LVEF, impairment in the myocardial performance index. and contractile reserve.
De Jesus et al. [118]	2017	Anakinra (10 mg/kg)	Daily, starting 24 h after surgery	Anakinra improved conduction velocity and reduced action potential duration dispersion, thus determining a reduction of spontaneous and inducible ventricular arrhythmias.
Mauro et al. [119]	2017	IL-1α-blocking antibody (15 μg/kg)	Single dose after reperfusion	At 24 h, IL-1α blockade significantly reduced inflammasome formation and infarct size, thus preserving LVFS.
Herouki et al. [120]	2017	Anti-IL-1β Ab	Single dose after reperfusion or 7 days after reperfusion	Immediate, but not delayed, administration of anti-IL-1β Ab reduces ischemia/reperfusion-related infarct size, left ventricular remodeling, and heart-failure-related coronary dysfunction.

IL: interleukin; rhIL-1Ra: recombinant human interleukin-1 receptor antagonist; LVEF: left ventricular ejection fraction; LVFS: left ventricular fractional shortening.

**Table 2:** Clinical studies investigating the effect of IL-1 inhibition in preventing cardiac remodeling after myocardial infarction.

Author	Year	Drug	Treatment	Disease (cohort)	Results
Abbate et al. VCU-ART [128]	2010	Anakinra	100 mg/daily sc for 14 days	STEMI (n = 10)	In this pilot double blind RCT, treatment with anakinra showed to be safe and to reduce left ventricular remodeling (assessed by both echocardiography and cardiac magnetic resonance) after STEMI as compared to placebo.
Morton et al. MRC-ILA-HEART [129]	2015	Anakinra	100 mg/daily sc for 14 days	NSTEMI (n = 182)	In this proof-of-principle double blind RCT, patients treated with anakinra showed reduced levels of hsCRP and IL-6 as compared to those receiving a placebo.
Abbate et al. VCU-ART2 [121]	2013	Anakinra	100 mg/daily sc for 14 days	STEMI (n = 30)	In this pilot double blind RCT, treatment with anakinra could reduce hsCRP levels as compared to a placebo. Anakinra-treated patients also showed a numerically lower incidence of heart failure, although this was not statistically significant.
Ridker et al. CANTOS [125]	2019	Canakinumab	50, 100 or 150 mg/daily sc every 3 months	STEMI (n = 10’061)	In this double blind RCT, treatment with canakinumab after STEMI was shown to dose-dependently reduce hospitalization for heart failure and the composite of hospitalization for heart failure or heart-failure-related mortality as compared to a placebo.
Van Tassell et al. VCU-ART3 [130]	2019	Anakinra	100 mg once or twice/daily for 14 days	STEMI (n = 99)	Preliminary results of this double blind RCT were presented at the 2019 Congress of the European Society of Cardiology. Patients treated with anakinra showed significant improvement in cardiac systolic function after STEMI, as compared to a placebo.

CANTOS: Canakinumab Anti-Inflammatory Thrombosis Outcomes Study; hsCRP: high-sensitivity C-reactive protein; IL-6: interleukin-6; NSTEMI: non-ST-elevation myocardial infarction; RCT: randomized clinical trial; STEMI: ST-elevation myocardial infarction; VCU-ART: Virginia Commonwealth University Anakinra Remodeling Trial.
